# Epidemiological findings of major chemical attacks in the Syrian war are consistent with civilian targeting: a short report

**DOI:** 10.1186/s13031-018-0150-4

**Published:** 2018-04-16

**Authors:** Jose M. Rodriguez-Llanes, Debarati Guha-Sapir, Benjamin-Samuel Schlüter, Madelyn Hsiao-Rei Hicks

**Affiliations:** 1 Faculty of Economic, Social and Political Sciences and Communication, Université catholique de Louvain, Brussels, Belgium; 2 Centre for Research on the Epidemiology of Disasters, Institute of Health and Society, Université catholique de Louvain, Brussels, Belgium; 3 Department of Psychiatry, University of Massachusetts Medical School, Worcester, MA USA

**Keywords:** Syria, Conflict, War, Chemical weapons, Civilian, IHL, Mortality, Direct deaths

## Abstract

Evidence of use of toxic gas chemical weapons in the Syrian war has been reported by governmental and non-governmental international organizations since the war started in March 2011. To date, the profiles of victims of the largest chemical attacks in Syria remain unknown. In this study, we used descriptive epidemiological analysis to describe demographic characteristics of victims of the largest chemical weapons attacks in the Syrian war.

We analysed conflict-related, direct deaths from chemical weapons recorded in non-government-controlled areas by the Violation Documentation Center, occurring from March 18, 2011 to April 10, 2017, with complete information on the victim’s date and place of death, cause and demographic group. ‘Major’ chemical weapons events were defined as events causing ten or more direct deaths.

As of April 10, 2017, a total of 1206 direct deaths meeting inclusion criteria were recorded in the dataset from all chemical weapons attacks regardless of size. Five major chemical weapons attacks caused 1084 of these documented deaths. Civilians comprised the majority (*n* = 1058, 97.6%) of direct deaths from major chemical weapons attacks in Syria and combatants comprised a minority of 2.4% (*n* = 26). In the first three major chemical weapons attacks, which occurred in 2013, children comprised 13%–14% of direct deaths, ranging in numbers from 2 deaths among 14 to 117 deaths among 923. Children comprised higher proportions of direct deaths in later major chemical weapons attacks, forming 21% (*n* = 7) of 33 deaths in the 2016 major attack and 34.8% (*n* = 32) of 92 deaths in the 2017 major attack.

Our finding of an extreme disparity in direct deaths from major chemical weapons attacks in Syria, with 97.6% of victims being civilians and only 2.4% being combatants provides evidence that major chemical weapons attacks were indiscriminate or targeted civilians directly; both violations of International Humanitarian Law (IHL). Identifying and quantifying chemical weapons violations requires inter-disciplinary collaboration to inform international policy, humanitarian intervention and legal action.

## Background

The use in war of chemical weapons was condemned by the general opinion of the civilized world and prohibited with the signing of the 1925 Geneva Protocol [1]. Further prohibition includes the Convention on the Prohibition of the Development, Production, Stockpiling and Use of Chemical Weapons and on their Destruction (CWC), which became binding international law in 1997 [2]. In violation of these prohibitions, some parties to war have used chemical weapons in conflicts [3], including in Syria. Evidence of use of chemical weapons in Syria in the form of numerous toxic gas attacks has been reported by governmental and non-governmental international organizations since the Syrian war started in March 2011. Joint investigations by the Organization for the Prohibition of Chemical Weapons (OPCW) with the United Nations (UN) between April and November 2013 confirmed the use of chemical weapons in the Ghouta area of Damascus on 21 August 2013 and on a smaller scale, in Jobar on 24 August 2013, Saraqueb on 29 April 2013, Ashrafiah Sahnaya on 25 August 2013 and Khan Al Asal on 19 March 2013 [4]. Later reports provided evidence with a high degree of certainty that chlorine gas was used as a weapon in the villages of Talmenes, Al Tamanah, and Kafr Zita from April to August 2014 [5]. On 21 August 2015, the OPCW fact-finding mission (FFM) reported on the investigation of several incidents in the Idlib governorate between 16 March 2015 and 20 May 2015 and concluded that these gas attacks likely involved the use of sulphur mustard and in occasions combined with chlorine [6]. The Syrian American Medical Society (SAMS) carried out parallel investigations involving the collection by medical staff of biological samples taken from the many patients treated after gas attacks and environmental samples collected from the area of attacks. SAMS identified the use of three toxic gases – Sarin, chlorine and mustard agents – and confirmed the findings of OPCW-UN investigations [7]. In April 4, 2017 missiles launched from air allegedly loaded with toxic chemical gases attacked the town of Khan Sheikhoun in Idlib, Syria. The Violations Documentation Center (VDC) reported at least 92 identifiable deaths by the time of this study [8] and others, even more [9]. The medical humanitarian NGO, Médecins Sans Frontières (MSF) concluded that at least two toxic gases, Sarin and chlorine, may have been used in these attacks, based on evidence from MSF medical teams supporting the emergency department of Bab Al Hawa hospital and other hospitals [10]. A joint FFM by the OPCW-UN reported the presence of Sarin or a Sarin-like substance in laboratory samples taken by the FFM team during the autopsies of three victims by this attack [11]. To the date of this study, the Joint Investigative Mechanism (JIM) of the OPCW/UN, based on the FFM reports, concluded that the Syrian Air Force was the perpetrator of at least three attacks with chlorine (Kafr Zita - 18 April 2014, Qmenas - 16 March 2015 and Binnish, 24 March 2015). It also concluded that the Islamic State of Iraq and the Levant (ISIS/ISIL) perpetrated at least one attack with mustard agent (Mare’e, 21 August 2015) [12]. Investigations and evidence of the use of chemical weapons in Syria reported by governmental and non-governmental international organizations are essential components of civilian protection.

The research community has so far contributed relatively little to civilian protection or to understanding the public health consequences of chemical weapons attacks in Syria [13–16]. In earlier studies we described patterns of direct deaths and victim characteristics caused by the range of weapons used in the Syrian war from the beginning of the war in March of 2011 through December 2016 [15, 16]. Here we use descriptive epidemiological analysis to address in detail the fundamental question of who have been the victims of the largest chemical weapons attacks in the Syrian war from March 18, 2011 to April 10, 2017.

## Methods

Data were obtained with permission from the VDC [8], a non-profit, non-governmental independent organisation that tracks and documents in the public domain war-related deaths from the beginning of the Syrian war. Direct deaths documented by the VDC are arguably a verifiable minimum; most other sources report higher numbers but are without a comparable verification protocol [17, 18]. Only the VDC systematically reports sex, age category (children versus adult), and victim’s status (civilian versus combatant) of each recorded direct death from the Syrian war, thereby making these data particularly useful for epidemiological analysis of the impact of weapons on the Syrian civilian population [15–18].

The VDC applies international standards for documentation of human rights violations by a ground network consisting of a minimum 30 internationally-trained field reporters [15, 16]. Field reporters collect data in three steps. First, initial information on one or more victims is gathered, mainly from hospitals, morgues, relatives of the victims, and media sources. Second, the initial report is further confirmed, where possible, by supporting information on victims such as videos or photographs. Third, key information missing on victims is actively investigated until completion of record. VDC registries are verified daily, updated with new information and records for each death: demographics, date, location, cause of death and civilian versus combatant status. The VDC identifies combatant deaths by an iterative process including multiple sources of information and validation. All those identified as combatants by VDC in our analyses had information on military rank or armed group (*n* = 26). Given that ascertaining information on victims in government-controlled areas is often challenging, as stated by others conducting studies on the Syrian war [16, 19], we analysed data from non-government-controlled areas. Our inclusion criteria were: conflict-related, direct deaths from chemical weapons attacks, occurring from March 18, 2011 to April 10, 2017, with complete information on the victim’s date and place of death, cause and demographic group (adult male, adult female, child). Chemical weapons were defined as toxic substances delivered in any form. This study focused on ‘major’ chemical weapons events, defined as those causing ten or more direct deaths thereby meeting the inclusion criteria set out by the authors of this study. For the purposes of this study, we defined children as individuals younger than 18 years of age, consistent with the Convention on the Rights of the Child [20]. Further details on the VDC dataset and data collection are described elsewhere [8, 15, 16].

## Deaths from major chemical weapons attacks

A total of 1206 direct deaths meeting inclusion criteria were identified in the dataset from all chemical weapons attacks regardless of size during the study period. Five major chemical weapons attacks were identified that caused direct deaths of ten or more individuals. The five major chemical weapons attacks resulted in a total of 1084 documented direct deaths recorded as of 10 April 2017 and meeting study criteria. As is apparent from Table 1, VDC data indicate that nearly all of those who died from the five largest chemical attacks in Syria were civilians. Civilians composed 97.6% (*n* = 1058) of direct deaths from major chemical weapon attacks and combatants only 2.4% (*n* = 26). This extreme disparity in effect on victims suggests that chemical weapons attacks in Syria were indiscriminate or targeted against civilians, both being violations of International Humanitarian Law (IHL). The extreme disparity is also consistent with arguments that chemical weapon attacks were not as much a war tactic against opponents as a terror and displacement strategy against Syrian civilians.

**Table 1:** Direct deaths of civilians and combatants attributed to major chemical weapons attacks in the Syrian war (March 18th, 2011 to April 10th, 2017)

Location (city/village, province)	Date	Adult Males n (%)	Adult Females n (%)	Children n (%)	Civilian deaths per attack n (%)	Deaths per attack, % of total deaths^a^ n (%)
Khan Al Asal, Aleppo	19–03-13	12 (54.5)	7 (31.8)	3 (13.6)	22 (100.0)	22 (1.8)
Sheikh Maqsoud, Aleppo	13–04-13	9 (64.3)	3 (21.4)	2 (14.3)	14 (100.0)	14 (1.2)
Ghouta, Damascus Suburbs	21–08-13	524^b^ (56.8)	282 (30.5)	117 (12.7)	897 (97.2)	923^**b**^ (76.5)
Jrouh, Hama	12–12-16	21 (63.6)	5 (15.2)	7 (21.2)	33 (100.0)	33 (2.7)
Khan Sheikhoun, Idlib	04–04-17	39 (42.4)	21 (22.8)	32 (34.8)	92^c^ (100.0)	92 (7.6)
Total		605^**b**^ (55.8)	318 (29.3)	161 (14.9)	1058 (97.6)	1084^**b**^ (89.9)

^a^All chemical weapons attacks caused a total of 1206 documented direct deaths. ‘Major’ chemical weapons attacks were defined as those causing ten or more direct deaths. ^b^Of which 26 deaths were of adult male combatants from the Free Syrian Army (FSA). ^c^Documented as of 10 April 2017. Source: VDC

Among the 1058 civilian direct deaths from major chemical attacks, civilian men carried the largest burden of death comprising over half of direct civilian deaths in the five major chemical attacks (*n* = 579, 54.7%), women 30.0% (*n* = 318) and children 15.2% (*n* = 161). Of the earliest four major chemical attacks, in 2013, two occurred in Aleppo (19 March 2013 and 13 April 2013) and one in Ghouta, Damascus suburbs (21 August 2013). In the 21 August 2013 attack in Damascus suburbs, which caused the largest number of 897 civilian deaths among the five major attacks, 30 % of the victims killed were women (Table 1). In the case of the Ghouta attack, independent investigations by OPCW/UN found positive for Sarin and Sarin signatures biological samples from various victims. The other two former attacks were field-investigated but were inconclusive on chemical used [4]. After 3 years, on 12 December 2016, Jrouh village in Hama was bombed by two shells filled with Sarin gas which is a lethal toxic gas. Over a fifth of the immediate deaths from this attack were of children (*n* = 7). As can be seen in Table 1, children comprised higher proportions of direct civilian deaths in the last two of the five major chemical attacks during the study period. In particular, the most recent major chemical attack, on 4 April 2017 in Khan Sheikhoun, Idlib, where Sarin or Sarin-like substances were used [11], caused the highest proportion of deaths of children, who composed one-third of direct deaths from this attack (*n* = 32, 34.8%). As was the case in four of the five major chemical weapons attacks during the study period, no combatants were documented dead from the attack (Table 1), a finding that supports arguments that IHL was violated with respect to the protection of civilians.

## Consequences of chemical attacks

Sarin and other nerve agents are organophosphorus compounds that attack the nervous system by interfering with degradation of the neurotransmitter acetylcholine at neuromuscular junctions. Death occurs from asphyxia due to loss of control and paralysis of muscles involved in breathing [3, 21]. Exposure to Sarin can be fatal through dermal contact, by breathing air containing Sarin or by consuming contaminated food or water [3, 21–23]. However this intoxication risk is short-lasted as Sarin does not persist more than a 4–8 days in the environment [22]. Chlorine gas is the elemental form of Chlorine and is a severe pulmonary, dermal as well as digestive irritant. Inhalation causes life-threatening respiratory distress and fluid accumulation in the lungs potentially leading to death by suffocation [7]. Sulfur mustard, commonly known as mustard gas, is a class of cytotoxic compounds. Sulfur mustard compounds can be severe dermal, conjunctival and pulmonary irritants, can cause severe burns and are carcinogenic as well as blistering agents [3, 6, 7].

All of these toxic gases are heavier than air, causing the chemical agents to sink to low-lying areas to create a greater exposure hazard [7, 23]. Because of this, civilians who shelter in basements, which typically offer greater protection from explosive attacks such as barrel bombs, are at a heightened risk of death from chemical attacks, such as from barrel bombs containing chlorine used in Syria [7]. Being closer to the ground, children are more exposed to chemical agents. In addition, small children are at especially high risk from chemical agents as they have a higher body-surface-area-to-mass ratio; skin that is more permeable, and high metabolic and respiratory rates [24–26]. The first reaction to bombing is to take shelter, but people are often unaware that they must also close doors, windows and air vents to prevent gas entering [7]. Populations subject to chemical attacks are usually uninformed on precautionary behaviors to reduce exposure and health consequences, pointing to the importance of public awareness campaigns such as those initiated in some areas of Syria [7]. Barriers that need to be overcome for effective public awareness campaigns in conflict areas can include disrupted infrastructure, population displacement, lack of educational resources, and limited communication and movement in an insecure environment.

## Recommendations: a call to advocacy and action

The UN Security Council (UNSC) and the world have condemned chemical attacks in Syria to little effect. In 2013, under President Bashar al-Assad, Syria became a State Party to the Treaty on the Chemical Weapons Convention as part of an agreement to acknowledge and relinquish its stocks of Sarin, Venomous Agent X, and mustard gas. Subsequently, use of nerve agents decreased, but was replaced by increased use of chlorine gas [7]. The UNSC Resolution 2209 condemned the use of chlorine gas as a weapon in Syria in 2015, yet chlorine attacks continued to be documented [7]. Regarding the destruction of the chemical weapons production facilities under the 2013 agreement, one hangar remains undestroyed and the condition of two other stationary aboveground facilities is unknown due to the security situation. In May 2016 the UNSC passed Resolution 2286 condemning attacks on medical facilities and personnel. In November 2016 Syrian pro-government forces carried out a chemical attack against medical personnel [27].

It is essential that impartial and credible investigations of these chemical weapon attacks continue by the OPCW-UN and NGOs and that a structure of IHL standards and prohibitions exists. However, as suggested by this course of events, if violations of IHL against using chemical weapons are allowed to continue with impunity, then offenders have no incentive to end the use of chemical weapons until their goals are achieved, as can be argued is occurring in Syria. For actors in conflict who find chemical attacks acceptable, moral condemnation by international bodies is ineffectual unless backed up by active prevention or consequences. Prevention by decreasing access to resources and components that are necessary to produce chemical weapons could potentially decrease the use of chemical weapons, but this can be difficult. For example, chlorine gas is relatively cheap to produce, is an agent commonly used in the chemical industry and is thus difficult to control [28]. We suggest urgent attention to the strict control of commercial sales of chlorine, for example by a regulatory authority, given that its use has become normative in the Syrian war as a weapon [7].

Increasing preparation, education and resources to deal with chemical attacks can decrease mortality and decrease the severity of injuries. Training of first response and medical personnel for appropriate responses to chemical attacks is an important measure to reduce casualties [3, 7, 29]. Despite great effort by medical and support personnel in Syria, training in Syria has been limited by the insecure environment, lack of supplies and severe overwork of the medical personnel caring for casualties of war [7, 29, 30]. Decontamination and protective equipment to minimize continued exposure to chemical agents is essential during rescue and treatment of chemical attack victims in order to reduce injuries and deaths [3, 7, 29]. Sarin is highly toxic even in very small amounts, and when absorbed through skin or respiratory channels can cause death within a few minutes to 2 h [3, 23]. Mustard agents produce the first symptoms in 2–24 h after exposure, have no antidote, cause long-term injuries and have a lower lethal capacity than Sarin-like substances [3, 6]. Due to lack of adequate protective equipment, especially in besieged areas, first responders and medical staff in Syria have sickened and died from chemical contamination while treating victims of attacks, and cross-contamination of patients occurs [7]. Personal Protection Equipment (PPE) kits for first responders and medical staff are an acute need [7, 29]. Severe shortages of antidotes such as atropine, ventilation equipment, protective equipment and means to clean or store contaminated clothing have increased chemical attack casualties in the Syrian war and continue to be critical unmet needs [7, 29, 30]. We urge increasing supplies of these basic, critical resources to medical personnel working in Syria in order to improve survival and in order to decrease the immediate and long-term impacts of chemical weapon attacks.

Long-term health effects of chemical exposure are not only generally unknown to local populations but can also last for years [3, 31]. The physical, neurological and mental health effects from chemical weapons exposure are both acute and long-term, requiring adequate resources for immediate treatment and for long-term effects. Some of the attacked areas are farming hamlets and are especially vulnerable to the potential contamination of food and the food chain by persistent agents such as mustard and nerve agents [28, 32]. Even if people are aware of the risks, frequently there are no alternative sources to meet their basic needs. Hence, attacks on rural regions with Sarin may have additional effects [31]. Having these facts in mind, risk communication, public awareness programs and monitoring for long-term effects are important public health measures to reduce the immediate and long-term burden of chemical attacks. The development of communications systems using social media and cell phone technology could provide widespread, rapid, public safety instructions, education and alerts for chemical weapons attacks [33, 34].

Epidemiological research on the public health impact of chemical weapons can increase awareness and information on the scope of effects on the population and can identify particularly vulnerable groups [13]. Such information can inform policy and interventions. Increased interdisciplinary communication and collaboration between researchers, personnel in the field and NGOs gathering data and experience may be especially important in addressing the impact of armed conflict on populations.

## Conclusions

We found that civilians comprised the vast majority of direct deaths from major chemical weapon attacks compared to combatants, to a degree that suggests that chemical weapon attacks in Syria were indiscriminate or targeted against civilians in violation of IHL. Children comprised higher proportions of direct deaths in the two most recent major chemical attacks of the study period, including in Khan Sheikhoun where they comprised one-third of all immediate deaths. The UNSC and other international bodies should not only investigate perpetrators of chemical attacks but also hold them accountable for their actions. Simultaneously, the international community needs to respond quickly with greater practical support to Syrian medical and emergency facilities so that they can meet the acute medical needs of a population targeted by chemical attacks.
